# Genetic Linkage Map Construction and QTL Mapping of Salt Tolerance Traits in Zoysiagrass (*Zoysia japonica*)

**DOI:** 10.1371/journal.pone.0107249

**Published:** 2014-09-09

**Authors:** Hailin Guo, Wanwen Ding, Jingbo Chen, Xuan Chen, Yiqi Zheng, Zhiyong Wang, Jianxiu Liu

**Affiliations:** Institute of Botany, Jiangsu Province and Chinese Academy of Sciences, Nanjing, China; Kansas State University, United States of America

## Abstract

Zoysiagrass (*Zoysia* Willd.) is an important warm season turfgrass that is grown in many parts of the world. Salt tolerance is an important trait in zoysiagrass breeding programs. In this study, a genetic linkage map was constructed using sequence-related amplified polymorphism markers and random amplified polymorphic DNA markers based on an F_1_ population comprising 120 progeny derived from a cross between *Zoysia japonica* Z105 (salt-tolerant accession) and Z061 (salt-sensitive accession). The linkage map covered 1211 cM with an average marker distance of 5.0 cM and contained 24 linkage groups with 242 marker loci (217 sequence-related amplified polymorphism markers and 25 random amplified polymorphic DNA markers). Quantitative trait loci affecting the salt tolerance of zoysiagrass were identified using the constructed genetic linkage map. Two significant quantitative trait loci (*qLF-1* and *qLF-2*) for leaf firing percentage were detected; *qLF-1* at 36.3 cM on linkage group LG4 with a logarithm of odds value of 3.27, which explained 13.1% of the total variation of leaf firing and *qLF-2* at 42.3 cM on LG5 with a logarithm of odds value of 2.88, which explained 29.7% of the total variation of leaf firing. A significant quantitative trait locus (*qSCW-1*) for reduced percentage of dry shoot clipping weight was detected at 44.1 cM on LG5 with a logarithm of odds value of 4.0, which explained 65.6% of the total variation. This study provides important information for further functional analysis of salt-tolerance genes in zoysiagrass. Molecular markers linked with quantitative trait loci for salt tolerance will be useful in zoysiagrass breeding programs using marker-assisted selection.

## Introduction

Soil salinity is an escalating problem worldwide that affects 10% of the land area of the world, which amounts to 1 billion hectares [1]. There is about 37 million hectares of land suffering from salinization and secondary salinization in China [2]. Worldwide, salinization continues to increase, particularly in arid and semiarid regions [1]. The growth of salt tolerant plants is an effective and economic way to reduce the spread of salinization and to make full use of land resources. Though most of this land is currently too saline for conventional agriculture, a good proportion of it has the potential to be used for growing salt tolerant grasses [3].

Zoysiagrass (*Zoysia* Willd.) is an important warm season turfgrass that is indigenous to countries on the western Pacific Rim and westward into the Indian Ocean [4]. There are 11 recognized species in this genus, of which five species of indigenous zoysiagrass, *Zoysia japonica*, *Z. matrella*, *Z. tenuifolia*, *Z. sinica*, and *Z. macrostachya*, have been identified in China [5]. Zoysiagrass is an allotetraploid species with 40 chromosomes [6], [7]. Numerous studies have assessed the effects of salinity on zoysiagrass growth and salinity tolerance [8]–[13]. Zoysiagrass is ranked as a salt tolerant turfgrass [14]; however, considerable variability in salt tolerance exists among zoysiagrass species and genotypes [11]–[13], [15]. Therefore, the characterization of its salt tolerance is important for developing *Zoysia* cultivars with high salt tolerance. In a conventional breeding program, it can be difficult to evaluate salt tolerance because the salt concentration in a field has a gradient in either a horizontal or vertical direction. Therefore, in breeding for salt tolerance, DNA marker-assisted selection is particularly useful to select inherited genetic markers that are associated with the trait, and to use them as indirect selection criteria for marker-assisted breeding.

A detailed genetic map is essential for a quantitative trait locus (QTL) analysis. Several genetic maps of zoysiagrass have been constructed using PCR-based markers, such as simple sequence repeats (SSRs) [16], [17], amplified-fragment length polymorphisms (AFLPs) [18], [19], and restriction-fragment length polymorphisms (RFLPs) [7], [20]. These genetic maps have provided a solid basis for the QTL analysis of important traits of zoysiagrass. For example, Yaneshita et al. [20] analyzed the QTLs associated with winter leaf color based on the RFLP linkage map, and Ding et al. [21] analyzed the QTLs associated with cold tolerance based on the SSR linkage map. However, no genetic map specific for any of the important salt tolerance traits in zoysiagrass has been constructed to date. In our previous studies, an F_1_ population of *Z. japonica* was developed from a cross between a salt-tolerant parent and a salt-sensitive parent. Each salt tolerance trait showed a continuous distribution across the F_1_ population. The genetic model analysis showed that some major genes for salt tolerance exist in zoysiagrass [22], [23].

SRAP (sequence-related amplified polymorphism) is a novel PCR-based marker technique, which aims to amplify open reading frames (ORF) with particular primer pairs [24]. It provides a unique combination of forward and reverse primers that can be selected arbitrarily, providing a large number of primer combinations. Because this is an ORF-based marker system, it targets functional genes and could be applied in crop breeding [25]. The SRAP marker system is a simple, efficient and reliable marker system that can be adapted for a variety of purposes, such as map construction, QTL mapping [26]–[30], comparative genetics [31], and genetic diversity assessment [32]. In 2008, we established and optimized the SRAP-PCR reaction system in zoysiagrass and applied this new technique to authenticate hybrids and analyze the diversity of genetic markers related to cold tolerance and the green period of zoysiagrass. The results showed that SRAP markers are useful and efficient for zoysiagrass [33]–[35].

Random Amplified Polymorphic DNA (RAPD) markers tend to estimate intra- or intergenetic distances among more distantly related individuals or closely related genotypes (4–6) [36], [37]. Despite many weaknesses, RAPD is relatively easy and fast, and provides a high degree of polymorphisms and a virtually inexhaustible pool of possible genetic markers, making the technique advantageous over other molecular techniques [37]. These benefits justify the frequent application of the technique. It has been developed and used extensively to assess genetic diversity, phylogenetic relationships, construct genetic maps and identify QTLs [36], [38]–[41]. Some genetic linkage maps of zoysiagrass have been constructed using AFLP, RFLP and SSR molecular markers [7], [16]–[18], [42]. However, RAPD markers have not been used. Adding some RAPD markers and incorporating other markers may be useful for constructing a high density linkage map for zoysiagrass.

In this study we developed a genetic linkage map for a population derived from an intraspecific cross between two *Z. japonica* germplasms (Z105 and Z061) using SRAP and RAPD markers, and reported a mapping of QTLs affecting salt tolerance. The results provide important information for further functional analysis of salt tolerance genes in zoysiagrass. The molecular markers linked with QTLs for salt tolerance could be used for breeding programs in zoysiagrass using marker-assisted selection.

## Materials and Methods

### Plant material and DNA isolation

An F_1_ population comprising 120 progeny developed from the intraspecific cross of Z105 (*Z. japonica*, salt tolerant accession) and Z061 (*Z. japonica*, salt sensitive accession) was used to construct genetic linkage maps. Z105 is a germplasm that was collected from the coastal region of Yantai, Shandong Province, China in 1999. It suffers NaCl injury when the salt concentration is equal to or greater than 4% [43]. Z061 was collected from the natural grassland of Nanjing, Jiangsu Province, China in 1995. It suffers NaCl injury when the salt concentration is equal to or greater than 0.5% [43]. All F_1_ lines and the two parents were planted into 7-cm-diameter ×8-cm-deep pots filled with a mixture of field soil and river sand (4∶1). The pots were placed in an experimental field of the Institute of Botany, Jiangsu Province and Chinese Academy of Sciences (32°02′N, 118°28′E; elevation 30 m). All F_1_ lines were identified previously to be true hybrids [22]. Genomic DNA was extracted from the fresh leaves of each accession using a Plant Genomic DNA Extraction Kit (Yuanpinghao Biotech Co. Ltd, Tianjin, China). The quality of the extracted DNA was verified by 1% agarose gel electrophoresis. The DNA samples were stored at −20°C.

### SRAP and RAPD profiling

Ninety-eight pairs of SRAP polymorphic primer combinations [24] and 29 10-mer RAPD polymorphic primers between the two parents were used to genotype the mapping population. SRAP-PCR was performed in a total volume of 10 µL containing 50 ng genomic DNA, 1 µL of 10× PCR buffer, 1.5 mmol·L^−1^ MgCl_2_, 200 µmol·L^−1^ dNTPs, 0.2 µmol·L^−1^ primers, and 0.5 U Taq DNA polymerase. PCR reactions were performed in a TC-412 PCR thermal cycler (Bibby Scientific, Stone, United Kingdom) under the following conditions: predenaturation at 94°C for 4 min; followed by five cycles of denaturation at 94°C for 1 min, annealing at 37°C for 1 min, and extension at 72°C for 10 s; then 35 cycles of denaturation at 94°C for 1 min, annealing at 50°C for 1 min, and extension at 72°C for 10 s; and then a final extension at 72°C for 7 min. The PCR products were stored at 4°C before being separated on 10% non-denaturing polyacrylamide gels in 1× TBE buffer (pH 8.0). The gels were stained with fast silver stain. RAPD-PCR was performed in a total volume of 20 µL containing 50 ng genomic DNA, 2 µL of 10× PCR buffer, 2.5 mmol·L^−1^ MgCl_2_, 190 µmol·L^−1^ dNTPs, 0.5 µmol·L^−1^ primers, and 1.0 U Taq DNA polymerase. Amplifications were performed under the following conditions: predenaturation at 94°C for 3 min; followed by 45 cycles of denaturation at 94°C for 45 s, annealing at 36°C for 30 s, and extension at 72°C for 1.5 min; and then a final extension at 72°C for 5 min. Amplification products were stored at 4°C before being electrophoresed through 1.2% agarose gels run at 120 V for 1.5 h in 1×TAE buffer, and visualized using a JS-380A automatic gel imaging analysis system (Shanghai, China). The SRAP primer sequences were the same as those in Zheng et al. [30]. The RAPD primer sequences are listed in Table 1.

**Table 1:** RAPD primer sequences used for polymorphism analysis in the F1 population of *Zoysia japonica.*

Primer	Sequence	Primer	Sequence	Primer	Sequence
**A11**	5′-CAATCGCCGT-3′	**G01**	5′-CTACGGAGGA-3′	**O15**	5′-TGGCGTCCTT-3′
**A19**	5′-CAAACGTCGG-3′	**H16**	5′-TCTCAGCTGG-3′	**OPQ11**	5′-TCTCCGCAAC-3′
**B01**	5′-GTTTCGCTCC-3′	**I16**	5′-TCTCCGCCCT-3′	**OPV4**	5′-CCCCTCACGA-3′
**B02**	5′-TGATCCCTGG-3′	**I18**	5′-TGCCCAGCCT-3′	**P01**	5′-GTAGCACTCC-3′
**B03**	5′-CATCCCCCTG-3′	**J14**	5′-CACCCGGATG-3′	**Q20**	5′-TCGCCCAGTC-3′
**B04**	5′-GGACTGGAGT-3′	**K18**	5′-CCTAGTCGAG-3′	**R03**	5′-ACACAGAGGG-3′
**B14**	5′-TCCGCTCTGG-3′	**M15**	5′-GACCTACCAC-3′	**V05**	5′-TCCGAGAGGG-3′
**C06**	5′-GAACGGACTC-3′	**N09**	5′-TGCCGGCTTG-3′	**V16**	5′-ACACCCCACA-3′
**E09**	5′-CTTCACCCGA-3′	**N17**	5′-CATTGGGGAG-3′	**X05**	5′-CCTTTCCCTC-3′
**F03**	5′-CCTGATCACC-3′	**O09**	5′-TCCCACGCAA-3′		

RAPD; random amplified polymorphic DNA.

### Genetic linkage map construction

JoinMap 3.0 [44] was used to construct the linkage map. Based on the JoinMap3.0 developers' definition, the F_1_ population in this study could be considered as a cross-pollination population because the genetic background of the two parents was heterozygous. Three segregation type codes <lm×ll>, <nn×np> and <hk×hk> were used to score heterozygous loci in the female parent (Z105), the male parent (Z061), and in both parents, respectively. For fragments that were heterozygous in only one of the parents (testcross markers), the segregation ratio across the mapping population was tested against a 1∶1 ratio using a *χ^2^* test, while fragments that were heterozygous in both parents (intercross markers) were tested against a 3∶1 ratio. Segregation of markers that did not fit either ratio (*P*<0.05) was treated as distorted. The recombination frequency was converted to a genetic map distance (Morgan, M) using the Kosambi mapping function [45]. Logarithm of odds (LOD) scores of 3.5 were used to determine all the linkage groups (LGs).

### Estimation of genome length

The expected size of the zoysiagrass genome was estimated using *∑L_i_*[(*k_i_*+1)/(*k_i_*−1)], as described by Chakravarti et al. [46], where *Li* is the length of the *i*th linkage group (cM), *k_i_* is the number of marker loci on the *i*th linkage group. Genome coverage was estimated using the ratio between the cumulative map length and the expected genome size.

### Evaluation of salt tolerance

The experiment was conducted from 20 July to 30 October 2010 in solution culture in a glasshouse. During the experiment, the daily minimum and maximum air temperatures in the greenhouse were 15 and 35°C, and the average air temperature was about 25°C. Plants were grown under natural light, with photosynthetically active radiation ranging from 800 to 1800 µmol·m^−2^·s^−1^. All F_1_ progeny and the two parents were planted from sod pieces into 9 cm-diameter, 6 cm-deep plastic pots having coarse nylon screen bottoms, and filled with coarse silica sand. Six pots (three for salinity treatment, three as control) were planted for every sample. Pots were suspended over tanks containing 45L (66.5×45.5×17.0 cm^3^) 1/2 Hoagland's solution as the nutrient medium. Grasses were clipped weekly and allowed to establish well before salinity treatments began. After 2 months of plant culture, salinity treatments were applied. To avoid salinity shock, salinity levels were gradually increased by increments of 2.5 g NaCl·L^−1^every day until the final treatment level in solution was 20 g NaCl·L^−1^. Immediately after the final treatment salinity levels were reached, shoots were clipped at 4 cm height, and the clippings discarded. Grasses were exposed to the salinity treatment for 1 month, and the leaf firing percentage (LF) of salinity-treated plants was determined by visual estimation of the total percentage of chlorotic leaf area. Grasses (all treatments and control) were clipped to a height of 4.0 cm, and dried at 70°C for 48 h for dry weight determination. LF and reduced percentage of dry shoot clipping weight (treatment to control, SCW) were used as the index to evaluate salt tolerance of all F_1_ progeny and the two parents.

Guo et al reported the salt tolerance results previously [22], [23]. Briefly, the parents and all their progeny had a slower growth rate under salt stress than the control, and the leaves in plants under salt stress turned yellow. The two parents differed markedly and the progeny displayed continuous segregation for the two traits. The LF was 35% for the female parent (Z105) and 95% for the male parent (Z061). The range of variation of LF for the progeny was 10–98% with an average of 50%. The SCWs of the two parents were 42% for Z105 and 85% for Z061. The range of variation of the SCW for the progeny was 2–91% with an average of 52%.

### QTL analysis

MapQTL 5.0 [47] was used to map the QTLs for LF and SCW using the MapQTL interval mapping method. LOD thresholds at a significance level of *P* = 0.05 were determined using 1000 permutations. A QTL was declared when the LOD value was higher than the threshold. The maximum LOD score in the interval determined the QTL positions. Confidence intervals associated with QTL locations were set as the map intervals corresponding to 1 LOD either side of the maximum LOD. The detected QTLs were located on LGs using the MAPChart software [48]. QTLs were named starting with ‘*q*’, followed by the abbreviated trait name (LF or SCW), and the number assigned to the QTL.

Additive(A) and dominant (D) effects of the QTLs were estimated from an output of the program MapQTL 5.0 as *A*_f_ = [(*µ*_ac_+*µ*_ad_)−(*µ*_bc_+*µ*_bd_)]/4 for female additivity, *A*_m_ = [(*µ*_ac_+*µ*_bc_)−(*µ*_ad_+*µ*_bd_)]/4 for male additivity, and *D* = [(*µ*_ac_+*µ*_bd_)−(*µ*_ad_+*µ*_bc_)]/4 for dominance, where *µ*_ac_, *µ*_ad_, *µ*_bc_, and *µ*_bd_ are the estimated phenotypic means associated with each of the four possible genotypic classes, ac, bc, ad, and bd, derived from a <ab×cd> cross [49]–[51].

## Results

### Polymorphism and segregation of markers in F1 population

Ninety-eight SRAP primer combinations with high polymorphism between the two parents were analyzed. A total of 293 polymorphic markers were generated in the F_1_ population, which comprised 120 F_1_ progeny of Z105 and Z061. The numbers of polymorphic markers per primer combination ranged from two to eight, with an average of three. Among the 293 polymorphic markers, 188 originated from Z105, 68 originated from Z061, and 37 existed in both parents and segregated in the F_1_ population (Table 2).

**Table 2:** Polymorphisms and segregation of the markers in the F_1_ population using JoinMap.

Segregation type	Segregation ratio						No. of markers^a^	No. of distorted markers^a^
	ll	lm	nn	np	h-	kk		
lmxll	1	1					188 (27)	24 (3)
nnxnp			1	1			68 (6)	24 (4)
hkxhk					3	1	37 (0)	7 (0)
Total							293 (33)	55 (7)

a The numbers indicate the number of sequence-related amplified polymorphism (SRAP) markers and the numbers in parentheses indicate the number of random amplified polymorphic DNA (RAPD) markers.

Twenty-nine RAPD primers were used to analyze the F_1_ population, and 33 polymorphic markers were identified. Only one or two polymorphic markers were scored per primer. Among the 33 polymorphic markers, 27 originated from Z105, six originated from Z061; however, no markers existed in both parents (Table 2).

The 326 polymorphic markers (293 SRAPs and 33 RAPDs) were fitted to the expected Mendelian segregation ratio using the *χ^2^* test at *P*<0.05: the markers specific only to either the female or male parent were fitted to a 1∶1 segregation ratio and the markers shared by both parents were fitted to a 3∶1 segregation ratio in the F_1_ population. Among all the markers scored, 264 (81%) showed a normal Mendelian segregation, with approximately 88.8% segregating at 1∶1 and the remaining 11.2% segregating at 3∶1. The 62 (19%) markers that did not follow a normal Mendelian segregation showed skewed segregations: 16.9% segregated from one parent and 2.1% segregated from both parents (Table 2). The skewed markers were included in the linkage map construction. Forty-four skewed markers were linked to LGs on the genetic linkage map; 23 were on LG1 and 21 were on the nine other LGs (Table 3).

**Table 3:** Map length, map density, and segregation distortion among the 24 linkage groups in the F_1_ population of *Zoysia japonica.*

Linkage group	Map length (cM)	No. of loci	Map density (cM/marker)	No. of loci with segregation distortion (*P*<0.05)	No. of inter-locus gaps (>20 cM)
1	163.0	48	3.4	23	1
2	142.3	45	3.2	4	0
3	205.2	23	8.9	5	0
4	56.0	19	3.0	1	0
5	44.1	11	4.0	2	0
6	17.9	11	1.6	0	0
7	75.9	9	8.4	2	0
8	63.9	12	5.3	1	0
9	43.3	11	3.9	2	0
10	47.3	9	5.3	0	1
11	40.5	7	5.8	0	0
12	29.9	5	6.0	0	0
13	31.2	4	7.8	1	0
14	23.1	3	7.7	0	0
15	32.1	3	10.7	0	1
16	28.3	4	7.1	0	1
17	28.0	3	9.3	0	1
18	8.1	3	2.7	0	0
19	14.6	2	7.3	1	0
20	33.4	2	16.7	1	1
21	11.0	2	5.5	0	0
22	3.3	2	1.7	0	0
23	38.8	2	19.4	1	1
24	29.7	2	14.9	0	1
Total/mean	1211	242	5.00	44	8

### Linkage map construction

**Figure 1:**
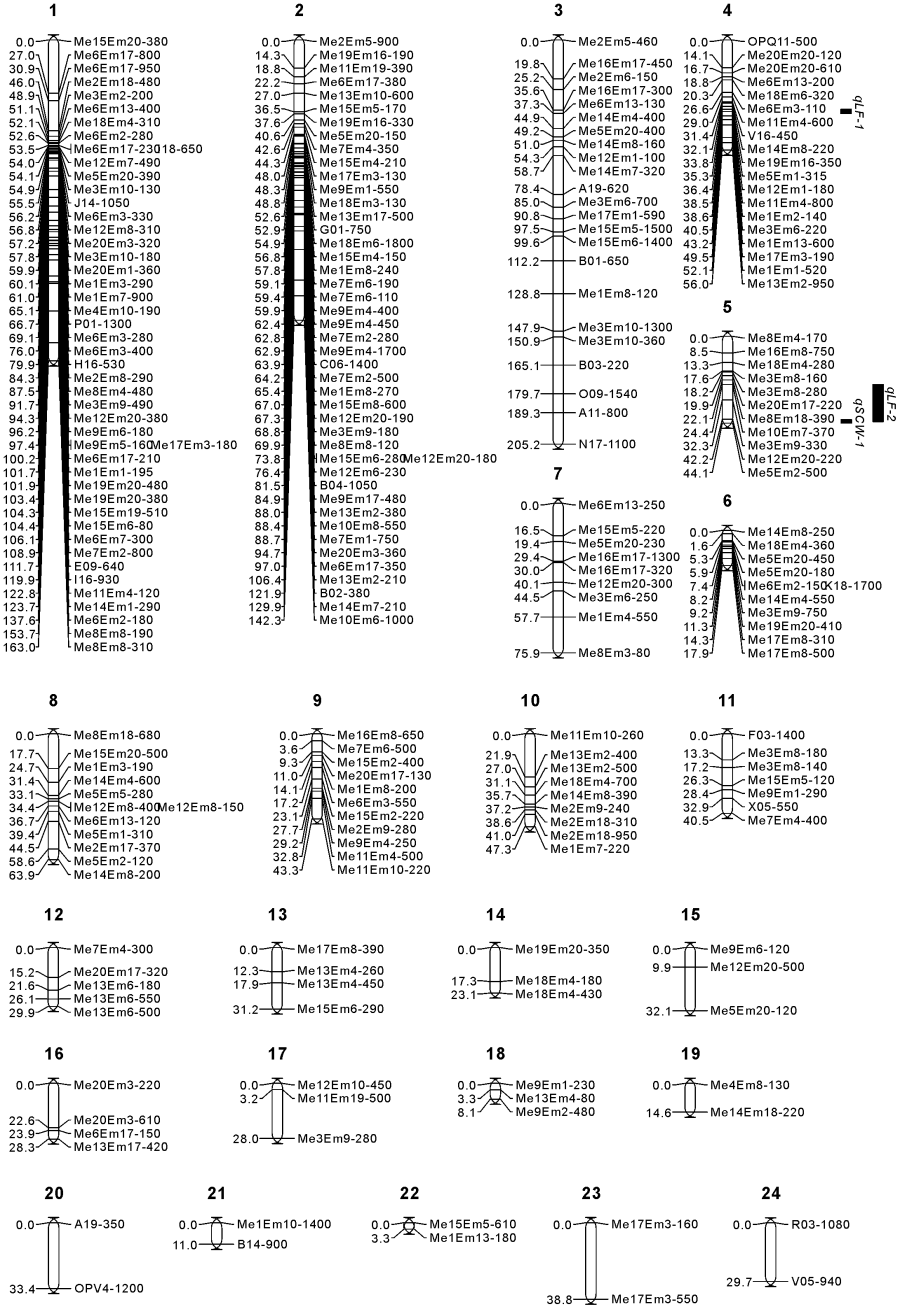
Linkage map from an F_1_ (Z105×Z061) population of *Zoysia japonica*, with 242 sequence-related amplified polymorphism (SRAP) and random amplified polymorphic DNA (RAPD) markers distributed on 24 linkage groups. The linkage map is 1211 cM long. Intervals in cM are shown on the left of each linkage group. The map was constructed using JoinMap3.0 with the Kosambi function and a logarithm of odds (LOD) threshold of 3.5. Quantitative trait locus (QTL) mapping was performed using the interval mapping method in MapQTL 5.0. The bars along the linkage maps indicate 1-LOD likelihood intervals for the QTLs. The QTLs are for the salt tolerance traits leaf firing percentage (*qLF*) and reduced percentage of dry shoot clipping weight (*qSCW*).

The 293 SRAPs and 33 RAPDs were used to construct the genetic linkage map; 217 of the SRAPs and 25 of the RAPDs were mapped into 24 LGs at the 3.5 LOD level. Based on the map lengths, the LGs were named LG1 to LG24. The 24 LGs contained 2–48 markers, and their map lengths ranged from 3.3 (LG22) to 205.2 cM (LG3). Altogether, the maps covered a total length of 1211 cM, with an average distance of 5.0 cM between markers. The gaps between markers ranged from 0 cM to 38.8 cM. The longest gap (38.8 cM) was between Me17Em3-160 and Me17Em3-550 on LG23, and gaps longer than 20 cM were located on LG1, LG10, LG15, LG16, LG17, LG20, LG23 and LG24 (Fig. 1). We estimated a total zoysiagrass genome size of 1733.8 cM using method 4 of Chakravarti et al. [46]. Based on this estimate of genome length, the 1211 cM total length of the constructed linkage map spanned 69.9% of the whole zoysiagrass genome.

### QTL mapping

The interval mapping method in MapQTL5.0 was used to analyze the QTLs for LF and SCW in zoysiagrass, based on the linkage map. Two significant QTLs for LF (*qLF-1* and *qLF-2*) were detected on LG4 and LG5, respectively. *qLF-1* explained 13.1% of the total variation of LF and was located at 36.3 cM on LG4, with a LOD value of 3.27, between markers Me5Em1-315 and Me12Em1-180,(1.0 cM from Me5Em1-315 and 0.1 cM from Me12Em1-180). *qLF-2* explained 29.7% of the total variation of LF and was located at 42.3 cM on LG5, with a LOD value of 2.88, in the same region as Me12Em20-220. *qLF-2* was 9.9 cM from Me3Em9-330 and 17.9 cM from Me10Em7-370 within the confidence interval. A significant QTL for SCW (*qSCW-1*) was detected in linkage group LG5. This QTL explained 65.6% of the total variation of SCW and was located at 44.1 cM on LG5, with a LOD value of 4.0, in the same region as Me5Em2-500. *qSCW-1* was 1.9 cM from Me12Em20-220 within the confidence interval.

Additive and dominant values revealed that all of female additive, male additive and dominant effects were found for these three QTLs. Female parent (Z105) had negative effects on *qLF-1* and *qLF-2* that resulted in reducing the leaf firing percentage. Male parent (Z061) had positive effects on *qLF-1* and *qLF-2* that resulted in increasing the leaf firing percentage. Both two parents had positive effects on *qSCW-1* that resulted in increasing the reduced percentage of dry shoot clipping weight. The overall dominance effects were negative for these three QTLs. The locations, nearest marker, percentage of the phenotypic variance explained by the QTL (PVE) and QTL directions of the three QTLs, are shown in Table 4 and Fig. 1.

**Table 4:** Characterization of the leaf firing percentage (LF) and reduced percentage of dry shoot clipping weight (SCW) QTLs for salt tolerance in zoysiagrass.

QTL	Linkage group	Position^a^ (cM)	Nearest marker	Confidence interval (cM)	LOD^b^	LOD^c^	PVE^d^ (%)	*A*_f_	*A*_m_	*D*
*qLF-1*	4	36.3	Me12Em1-180	35.3–37.4	3.27	3.0	13.1	−7.09	0.75	−1.60
*qLF-2*	5	42.3	Me12Em20-220	24.4–43.3	2.88	2.7	29.7	−3.22	10.38	−11.01
*qSCW-1*	5	44.1	Me5Em2-500	42.3–44.1	4.0	3.5	65.6	4.59	20.65	−3.16

a Position of the QTL (quantitative trait locus) peak on the corresponding LG.

b Maximum LOD (logarithm of odds) score (QTL peak).

c Threshold LOD score by permutation test.

d The percentage of the variance explained by the QTL.

*A*_f_ female additive effects, *A*_m_ male additive effects, *D* dominance effects.

## Discussion

### Marker polymorphism

In this study, we found that the SRAP markers detected more polymorphisms in zoysiagrass than the RAPD markers. Previously, we used SRAP markers to analyze the diversity of 96 *Zoysia* germplasm resources. Fifty-four SRAP primer combinations amplified a total of 337 polymorphic bands and the average number of polymorphic markers per primer combination was 6.7 [35]. These results showed that SRAP molecular markers are useful and informative markers for zoysiagrass.

In the F1 population in this study, 188 of the 293 SRAP markers and 27 of the 33 RAPD markers were detected in the maternal parent (Z105), while only 68 SRAP and 6 RAPD markers were found in the paternal parent (Z061), showing that Z105 contained many more polymorphisms than in Z061.This difference may be attributed to the different selfing ratio of zoysiagrass. A high variance range (0–72%) in the selfing ratio among different zoysiagrass samples has been observed previously [6], [52]. It was proposed that a high selfing ratio might reduce the level of polymorphisms [18]. Ebina et al. [19] amplified an interspecific two-way pseudo-testcross mapping population from a cross between *Z. japonica* and *Z. matrella* using AFLP markers, and detected only 31 polymorphisms in *Z. japonica*, and 188 in *Z. matrella*. To determine whether a high selfing ratio results in a low level of polymorphisms requires further study of the relationship between the selfing ratio and polymorphisms using more zoysiagrass samples.

### Segregation distortion

Segregation distortion has been reported in most mapping studies of plants [53]–[56], and high levels of segregation distortion have been reported in zoysiagrass. Cai et al. [16], [18] detected 10.8% segregation distortion of AFLP markers and 6% segregation distortion of SSR markers in an S_2_ population of *Z. japonica*. Li et al. [17], [42] detected 8.7% segregation distortion of SSR markers in a *Z. japonica* map and 43.7% segregation distortion of SSR markers in the *Z. japonica*×*Z. matrella*. Yaneshita et al. [7] detected 9.4% segregation distortion of RFLP markers in a *Zoysia* F_2_ population. In this study, 19% (62 of 326) of the SRAP and RAPD markers showed significant segregation distortion, which, based on the results described above, is normal for this F_1_ population. The distorted loci were distributed on 12 LGs; however, more markers with segregation distortion were observed in LG1 (23/62) and many of the skewed markers were located towards the end of LG1. This result is similar to a previous report [18] that found that many of the distorted AFLP markers were located at the end of LG2. In the previous studies discussed above, different markers were used to analyze different zoysiagrass populations, and in every one of these studies, some skewed markers were found. This observation may further indicate that the segregation distortion found in the present study was more likely to have been caused by genetic effects than by the population structure or the marker types [55].

### Genetic linkage map

The genetic linkage map between the two *Z. japonica* accessions presented in this study is a new map. The linkage map covers 69.9% of the estimated genome length, and has a total length of 1211 cM, a mean inter-marker distance of 5 cM, and contains 217 SRAP and 25 RAPD marker loci arranged on 24 LGs. The AFLP-based linkage map has a total length of 932.5 cM [18], and the integrated map of SSR and AFLP markers has a total length of 1187 cM [16], which makes them shorter than the new linkage map. However, the map based on RFLP markers (1506 cM) [7] and the maps based on SSR markers [17], [42] are longer.

The markers were not evenly distributed among the 24 linkage groups in our study, and the lengths of the LGs were quite different. Two LGs (LG1 and LG2) had more than 40 markers, while some LGs (LG19, LG20, LG21, LG22, LG23, and LG24) had only two markers. The longest LG (LG3) was 205.2 cM and the shortest was 3.3 cM. Some small LGs have been reported in zoysiagrass [7], [18], [19]. Li et al. [17] constructed a high-density SSR marker-based linkage map for *Z. japonica*; however, some small LGs with seven or eight markers were still detected. Similar results have been reported for other species [30], [56]–[58]. These observations might result from the nonrandom distribution of loci, the lack of marker polymorphisms on some chromosomes between mapping parents, or the shortage of available markers in corresponding regions of the chromosomes [59], [60]. Gaps that divide a chromosome into several LGs might be another explanation [58], because additional anchor markers would be needed to join the separated LGs.

In theory, the number of LGs should be equal to the number of haploid chromosomes. However, the numbers of LGs detected in this study and in the previous studies [7], [16]–[18], [42] are larger than the number of zoysiagrass chromosomes (20). This finding might be related to the type of mapping population, the number of individuals in the population, or the type and number of markers used to construct the map. To try to match the number of LGs to the number of chromosomes, more markers of different types could be used and incorporated with other markers in the publicly available linkage maps.

### QTL mapping

To help breeding of salt tolerant plants, a useful approach is to select simply inherited genetic markers that are associated with salt tolerance, and use them as indirect selection criteria for assisted breeding. QTLs associated with salt tolerance have been studied widely in many species [61]-[66], but not in zoysiagrass. In this study, crossing a salt-tolerant parent with a salt-sensitive parent produced the segregating population for salt tolerance in zoysiagrass. QTLs related to salt tolerance were analyzed, and three major QTLs on LG4 and LG5 were identified (Table 4, Fig. 1). Of the three QTLs, two (*qLF-1* and *qLF-2*) were related significantly to LF and explained 13.1 and 29.7% of the total variation, one (*qSCW-1*) was related significantly to the SCW and explained 65.6% of the total variation. These QTLs may be useful for marker-assisted breeding in zoysiagrass. However, three QTLs are not enough for salt tolerance breeding. More markers and a high density linkage map will be needed to identify salt tolerant QTLs for other traits, such as ion regulation and osmotic regulation. In addition, more QTLs for salt tolerance would be very useful for fine mapping or to isolate the most common salt-tolerance genes in zoysiagrass.

LF and SCW are two commonly used indices for salt tolerance of turfgrass. LF captures the survival rate of leaves and SCW indicates the growth rate of turfgrass under salt stress. In this study, both traits were used to evaluate salt tolerance in 120 progeny and two parents of zoysiagrass. Based on the phenotypic data, some plants had high LF and low SCW, while others had low LF and high SCW. The two parents are examples of these phenotypes. Other plants had either high LF and relatively high SCW, or low LF and relatively low SCW (data not shown). Clearly, a high LF does not always accompany a low growth rate under salt stress. Based on the QTL mapping, *qLF*-1 mapped on LG4, while two QTLs, *qLF-2* and *qSCW-1*, mapped close together on LG5; *qLF-2* at 42.3 cM and *qSCW-1* at 44.1 cM with confidence intervals of 24.4–43.3 cM and 42.3–44.1 cM, respectively. The confidence intervals of these two QTLs partly overlap. A detailed QTL analysis with more DNA markers is necessary to determine whether this is a single locus with a pleiotropic effect on the LF and SCW traits, if the same gene controls two traits simultaneously, or different genes control only one of the traits.

In conclusion, we have constructed a linkage map based on SRAP and RAPD molecular markers, and used it to identify QTLs related to LF and SCW for salt tolerance. DNA markers closely associated with the QTLs may be useful candidate markers for marker-assisted selection to improve salt tolerance in zoysiagrass.
